# Burden of intestinal parasitic infections and associated factors among pregnant women in East Africa: a systematic review and meta-analysis

**DOI:** 10.1186/s40748-023-00150-8

**Published:** 2023-04-06

**Authors:** Getu Mosisa, Dereje Chala Diriba, Reta Tsegaye, Gemechu Kejela, Diriba Bayisa, Adugna Oluma, Bizuneh Wakuma, Muktar Abadiga, Ebisa Turi, Tesfaye Abera, Lami Bayisa, Girma Tufa

**Affiliations:** 1 School of Nursing and Midwifery, Institutes of Health Sciences, Wollega University, Nekemte, Ethiopia; 2 Department of Public Health, Institutes of Health Sciences, Wollega University, Nekemte, Ethiopia; 3 Deakin Health Economics, Institute for Health Transformation, Deakin University, Geelong, VIC Australia; 4 Department of Midwifery, Institutes of Health Sciences, Bule Hora University, Bule Hora, Ethiopia

**Keywords:** Intestinal parasitic infection, Pregnant women, Associated factors, East Africa

## Abstract

**Background:**

The ultimate goal of preventing intestinal parasites among pregnant women is to reduce maternal and newborn morbidity and mortality. Numerous primary studies were conducted in East Africa presented intestinal parasite infection and associated factors among pregnant women. However, the pooled finding is not known. Therefore**,** this review aimed to identify the pooled prevalence of intestinal parasite infection and its determinants among pregnant women in East Africa.

**Methods:**

Articles published from 2009 to 2021 were searched in PubMed, Web of Science, EMBASE, and HINARI databases. The search for unpublished studies such as thesis and dissertations was checked in Addis Ababa University and Africa Digital Library. PRISMA checklist was used to report the review. Articles published in the English Language were considered. The data were extracted by two authors using data extraction checklists on Microsoft excel. Heterogeneity among the included studies was checked using I^2^ statistics on forest plots. Sensitivity and sub-group analyses were conducted to assess the presence of primary studies, and study characteristics responsible for the observed heterogeneity.

**Results:**

Of the 43 identified articles, about 23 articles were removed due to duplications. Then, by assessing the abstracts and full texts, four articles were removed because they failed to meet the eligibility criteria. Finally, 16 articles were included in the systematic and meta-analysis.The pooled prevalence of intestinal parasites among pregnant women in East Africa was 38.54 (28.77, 48.32). In this study, variables like residing in rural areas (OR: 3.75; CI: 1.15,12.16), availability of latrine(OR: 2.94; 95% CI: 2.22, 3.91), eating raw fruits/vegetables (OR: 2.44; 95% CI: 1.16, 5.11). and sources of water as unprotected sources (OR: 2.20; 95% CI: 1.11,4.35) show statistically significant association with the increased burden of intestinal parasites among pregnant women.

**Conclusion:**

The burden of intestinal parasite infection among pregnant women in East Africa was high. Therefore, efforts should be made in deworming pregnant women at the community and institutional level by stakeholders to reduce the burden of intestinal parasite infections and related complications.

## Background

Intestinal parasitic infections are mainly Soil-transmitted helminths which is a group of parasitic diseases caused by nematode worms that are transmitted to humans by fecal contaminated soil. The soil-transmitted helminths of major concern to humans are Ascaris lumbricoides, Trichuris trichiura, Necator americanus, and Ancylostoma duodenale [1]. Globally, about 24% of the world’s population is infected with soil-transmitted helminth infections, and sub-Saharan Africa areas are highly affected by the infection [2].

Studies showed that there is a high prevalence of intestinal parasitic infections in pregnant women, especially in some low- and middle-income countries[3]. Lack of proper sanitation and hygiene, the habit of eating raw vegetables, walking barefoot, and water sources are the major identified factors associated with soil-transmitted helminthic infections [4]. Pregnancy drains the body physically, physiologically, and immunologically, and together with intestinal parasitic infection, it may lead to fatal outcomes. Intestinal parasitic infections in pregnancy have been associated with serious adverse outcomes such as anemia, low birth weight, and mother and fetus morbidity and mortality [5–8].

World Health Organization urged endemic countries to start seriously tackling worms, specifically schistosomiasis and soil-transmitted helminths. It recommends periodic medicinal deworming without a previous individual diagnosis to all at-risk people, such as adolescents and pregnant and lactating women living in endemic areas, to achieve the 2030 global target of eliminating soil-transmitted helminths [2]. However, recent studies conducted in different parts of East Africa showed that the prevalence and impacts of these parasites are high among pregnant women [9–11].

Despite these deleterious effects on pregnant women, fetuses, and newborns in East Africa, the pooled prevalence, and associated risk factors of intestinal parasitic infections among pregnant women are not well explained. Numerous primary studies were conducted to assess the burden of intestinal parasites among pregnant women across East African countries. However, the pooled prevalence and factors associated with intestinal parasite is not identified. Therefore, this systematic review and meta-analysis aimed to assess the pooled prevalence of intestinal parasite infection and associated factors among pregnant women in East Africa.

## Methods

### Search strategies

Both published and unpublished articles were extensively searched for databases that include PubMed, Web of Science, EMBASE, HINARI, and Google Scholar.The search for unpublished studies such as thesis and dissertations was checked in Addis Ababa University and Africa Digital Library. The literature search was for the articles published from 2009 to 2021. Searches were conducted using terms such as “ Prevalence”, “Magnitude”, “Intestinal parasite”, “Intestinal helminths”, “Soil-transmitted helminths”, “Parasitic infection”, Pregnant women”, “associated factors”, “determinants” and all lists of East African countries “ by using Boolean operators like “ AND”and “OR”. Preferred Reporting Items for Systematic Reviews and Meta-Analyses (PRISMA) checklist and flow diagram were used for reporting the procedure.

### Selection and eligibility criteria

This systematic review and meta-analysis included studies that were conducted on intestinal parasite infection and associated factors among pregnant women in East Africa. The contents of each article were independently reviewed by two investigators (GM & DB) and finally articles that fulfilled the following criteria were included in the study. The study populations were all pregnant women living in East Africa. The review included all observational study designs reporting intestinal parasite infection among pregnant women in East Africa. Articles reported in the English language were included. We excluded articles that were not fully accessible, after at least two email contact attempts with the primary authors.

### Outcome measurement

This review considered two main outcomes. Intestinal parasite infection among pregnant women was the primary outcome of this study. It is measured as the total number of intestinal parasite infection cases over a total number of all women multiplied by 100. The second outcome of this study was to identify factors associated with intestinal parasites among pregnant women. For the second outcome, we determined the association between intestinal parasites and associated factors in the form of the log odds ratio. Residence (urban versus rural), availability of latrine (absence versus presence), the habit of eating raw fruit/vegetables (eating versus not eating), educational status (no education/primary education versus secondary and above), barefooted (Yes versus no), source of drinking water (protected versus unprotected), a habit of eating soil (absence versus presence) and hand washing after toilet (absence versus presence) were factors including as the determinants of intestinal parasite infection.

### Data extraction and quality assessment

In this review, the quality of the included studies was assessed using the Joanna Briggs Institute (JBI) Critical Appraisal Checklist for Analytical Cross-Sectional Studies [12]. Reference management software (Endnote version X7.2) was used to combine search results from databases and to remove duplications. Studies were screened using abstracts and titles. Then, the eligibility of the studies was evaluated using predetermined inclusion and exclusion criteria. The data were extracted by two authors (GM and DB) using data extraction checklists on Microsoft excel. For the outcome, intestinal parasite infections and associated factors, data were extracted in a format of two by two tables and then the log odd ratio was calculated based on the findings of the primary studies. The checklist for data extraction contains the author's name, year of publication, country, study design, sample size, response rate, and the number of participants with the outcome. Disagreement between two independent reviewers was resolved by involving the third reviewer (GK).

### Statistical analysis

After data extraction from original articles, data were exported to STATA version 14 for analysis. Heterogeneity among the included studies was checked using the Cochran *Q* test (chi-squared statistic) and I^2^ statistic on forest plots Sensitivity and sub-group analyses were conducted out to assess the presence of primary studies and study characteristics responsible for the observed heterogeneity.Heterogeneity was observed for the first outcome; therefore, a random-effects model was used to determine the pooled prevalence of intestinal parasite infection. A funnel plot, Egger’s tests were computed to test the presence of publication bias.

## Results

### Study selection

**Fig. 1:**
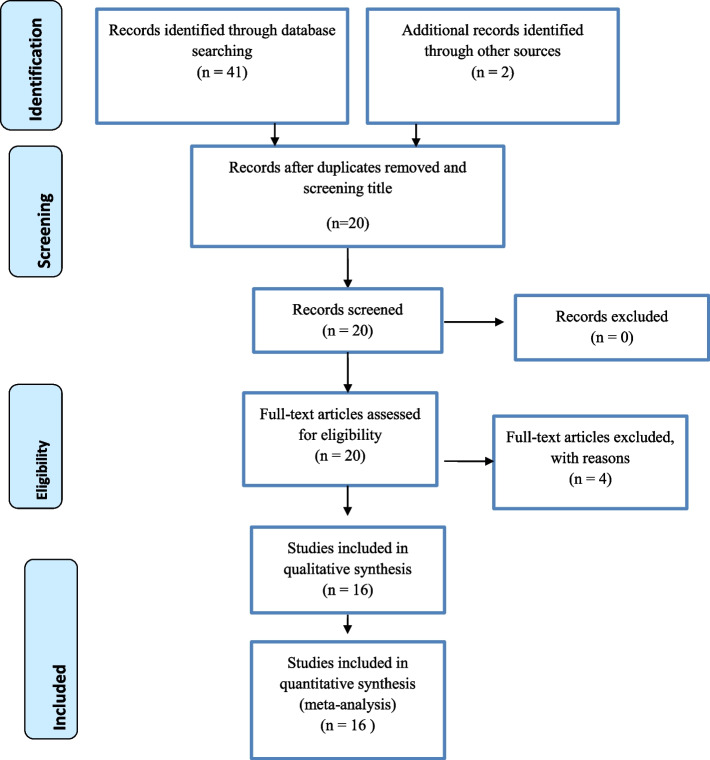
PRISMA flow diagram of included studies for the systematic review and meta-analysis of intestinal parasite infection and associated factors among pregnant Women in East Africa, 2021

A total of forty-three articles were identified through database and library catalog search. Of the identified articles, about 23 articles were removed due to duplications. Then, by assessing the abstracts and full texts, four articles were removed because they failed to meet the eligibility criteria. Finally, 16 articles that scored seven and above on the JBI quality appraisal were included in the systematic review and meta-analysis. Preferred Reporting Items for Systematic Reviews and Meta-Analyses (PRISMA) flow diagram were used to present the systematic review process (Fig. 1).

### Characteristics of included studies

A total of 4881 study partcipantswere involved in this systematic review. All included studies were cross-sectional. The sample size ranges from a minimum of 153 which was conducted in Kenya [13] to a maximum of 980 which was conducted in Rwanda [14]. Of the included studies, twelve of them were conducted in Ethiopia, two conducted in Kenya, one conducted in Uganda, and one conducted in Rwanda (Table 1).

**Table 1:** Summary of included studies on the prevalence of intestinal parasite infection and associated factors among pregnant women in East Africa, 2021

Author	Year of publication	Country	Study design	Sample size	Prevalence (95%CI)
Sewnet W et al. [14]	2020	Ethiopia	Cross-sectional	306	31.37(26.17,36.57)
Hylemariam M et al. [15]	2017	Ethiopia	Cross-sectional	372	24.73(20.35,29.12)
Adane D et al. [16]	2016	Ethiopia	Cross-sectional	384	31.51(26.86,36.16)
Million G et al. [17]	2013	Ethiopia	Cross-sectional	388	40.98(36.09,45.87)
Tadesse H et al. [18]	2020	Ethiopia	Cross-sectional	743	37.28(33.80,40.76)
Dawit J et al. [3]	2015	Ethiopia	Cross-sectional	258	29.46(23.89,35.02)
Wekesa et al. [12]	2014	Kenya	Cross-sectional	153	13.73(8.27,19.18)
Berhanu E et al. [8]	2018	Ethiopia	Cross-sectional	783	70.63(67.44,73.82)
Menasbo G et al. [19]	2019	Ethiopia	Cross-sectional	448	51.12(46.49,55.74)
Felister A et al. [20]	2020	Uganda	Cross-sectional	346	10.98(7.69,14.28)
Amelo B et al. [5]	2019	Ethiopia	Cross-sectional	349	38.68(33.57,43.79)
Demelash W et al. [10]	2021	Ethiopia	Cross-sectional	351	45.87(40.66,51.08)
Emil Ivan etal [13]	2013	Rwanda	Cross-sectional	980	34.29(31.31,37.26)
Girum Tefera [21]	2014	Ethiopia	Cross-sectional	748	58.29(54.76,61.82)
Shiferaw et al. [22]	2017	Ethiopia	Cross-sectional	180	21.11(15.15,27.07)
Anna M etal [23]	2009	Kenya	Cross-sectional	390	76.15(71.92,80.38)
Over all with weights from random effect(I^2^ = 98.8%, *P* = 0.000					38.54(28.77,48.32

### Pooled prevalence of intestinal parasite among pregnant women

**Fig. 2:**
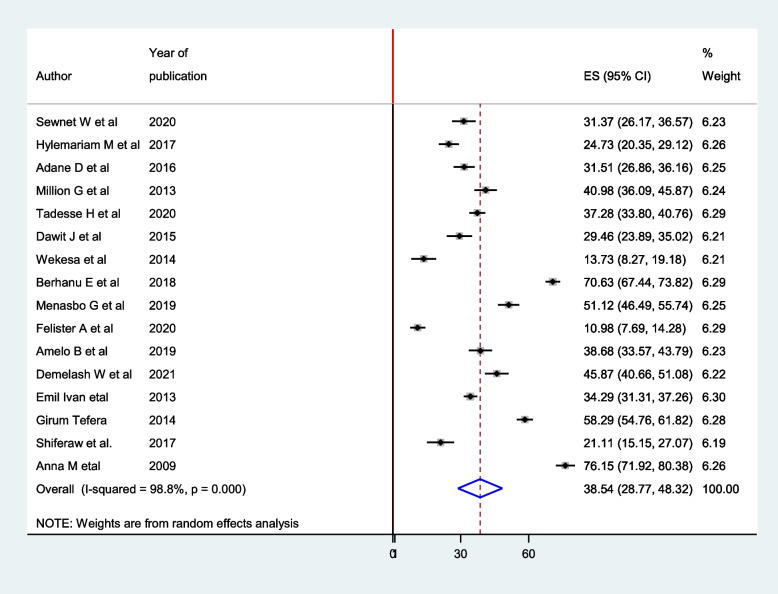
Forest plot for pooled prevalence of intestinal parasite among pregnant Women in East Africa, 2021

In this review, the highest prevalence of intestinal parasites was observed among pregnant women in Kenya (76.15 (71.92, 80.38), while the lowest 10.98 (7.69, 14.28) in Uganda. In this study, high heterogeneity was observed (I^2^ = 98.8 and *P*-value < 0.001), and therefore random effect model was used to assess the pooled prevalence of intestinal parasites among pregnant women in East Africa. Accordingly, the pooled prevalence of intestinal parasites among pregnant women in East Africa was 38.54 (28.77, 48.32) (Fig. 2).

### Sub-group analysis and publication bias

**Fig. 3:**
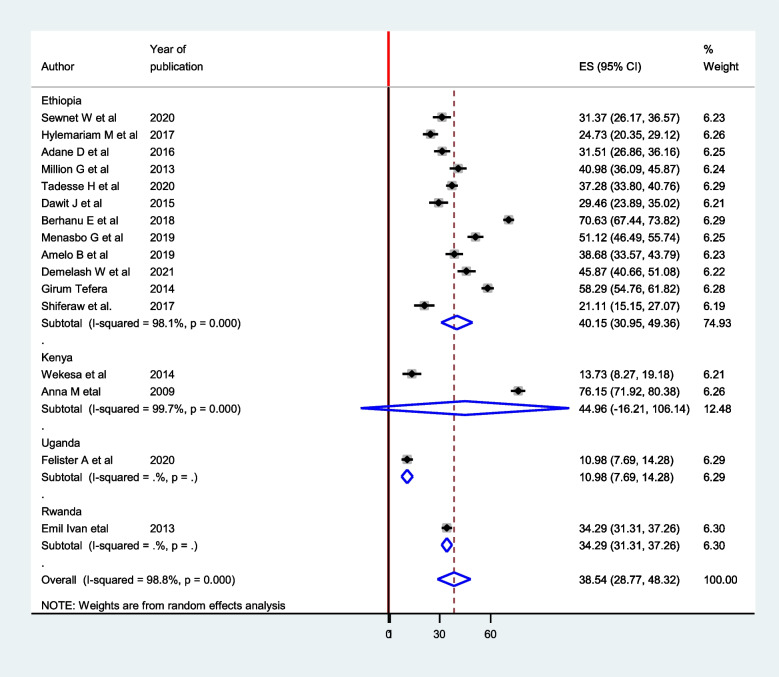
Subgroup analysis based on the country of prevalence of intestinal parasite among pregnant Women in East Africa, 2021

**Fig. 4:**
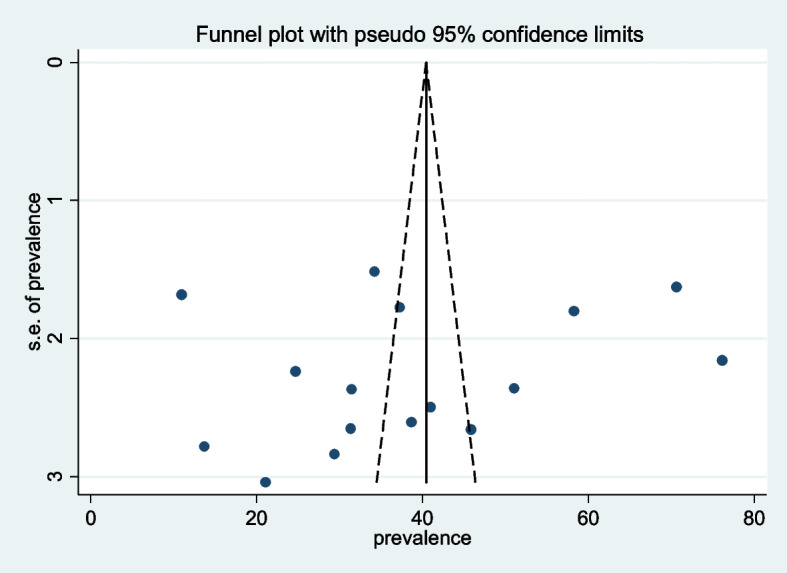
Funnel plot with 95% confidence limits of the pooled prevalence of intestinal parasite among pregnant Women in East Africa, 2021

In this meta-analysis, subgroup analysis was conducted using the country. The subgroup analysis showed that the prevalence of intestinal parasites among pregnant women was 40.15(30.95, 49.36) in Ethiopia [4, 6, 9, 11, 15–22], 44.96(-16.21, 106.14) in Kenya [13, 23], 34.29(31.31, 37.26) in Rwanda [14], and 10.98(7.69, 14.28) in Uganda [24] (Fig. 3). To assess the presence of publication bias, the funnel plot, and Egger test at 5% significant level were computed. The statistical test of the egger’s test indicated that there is no publication bias (*P* = 0.449). However, the visual interpretation of the funnel plot figure looks asymmetrical (Fig. 4).

### Sensitivity analysis

**Fig. 5:**
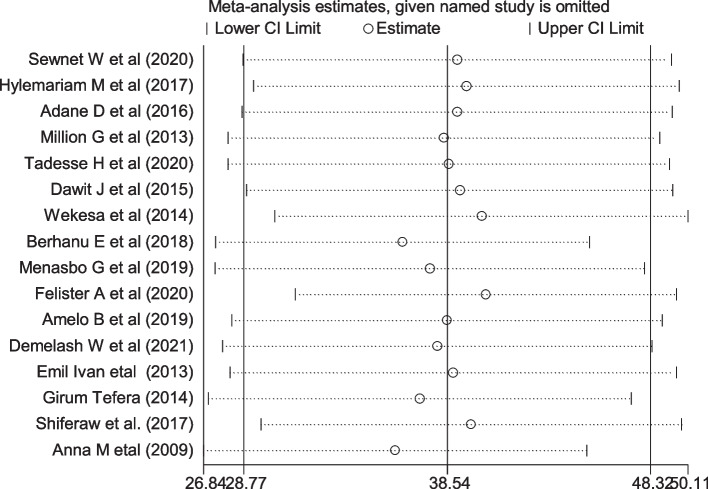
Sensitivity analysis of the prevalence of intestinal parasite infection among pregnant women of East Africa, 2021

Sensitivity analysis was conducted to identify the single study influence on the overall meta-analysis using a random effect model. The result showed that there was no strong evidence for the effect of single study influences on the overall meta-analysis (Fig. 5).

### Factors related with intestinal parasite among pregnant women

#### Association between residence and intestinal parasite

To assess the association between the intestinal parasite infection and residence, six articles [4, 9, 15–17, 19] were included. According to the finding, the rural residence was positively associated with the presence of the intestinal parasite. The pooled effect of the meta-analysis showed the positive association between rural residence and intestinal parasites among pregnant women.

**Fig. 6:**
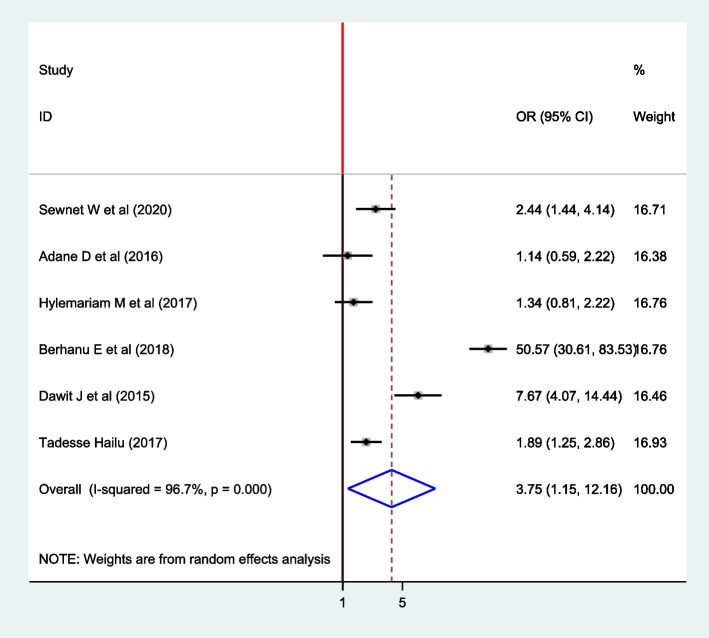
The forest plot for pooled association between intestinal parasite infection and residence among pregnant women in East Africa, 2021

The pooled finding indicated that the odds of developing intestinal parasite infection were 3.75 times higher among women who reside in the rural than their counterparts (OR: 3.75; CI: 1.15, 12.16). The random model effect model was used as heterogeneity was observed (I^2^ = 96.7, *P* =  < 0.001) (Fig. 6).

### Association between the availability of latrine and Intestinal parasite

**Fig. 7:**
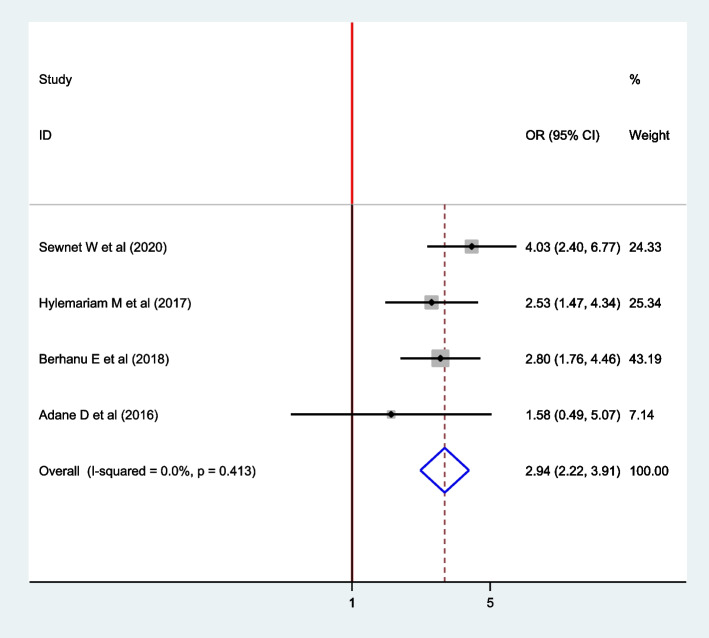
The forest plot for the pooled association between intestinal parasite infection and availability of latrine among pregnant women in East Africa, 2021

Four articles [9, 15–17] were included to assess the association between availability latrine and intestine parasite. Three articles showed the association between latrine and intestinal parasites [9, 15, 16] and one article did not show the association [17]. The study result indicated that the absence of latrine was associated with intestinal parasite among pregnant women. The pooled effect of the meta-analysis showed that the absence of latrine is positively associated with intestinal parasite infection. Pregnant women who have no latrine were 2.94 times more likely to have intestinal parasite compared to women who have latrine (OR: 2.94; 95% CI: 2.22, 3.91). A Fixed model was used since heterogeneity was not exhibited (I^2^ = 0.0%, *P* = 0.413) (Fig. 7).

### Association between a habit of eating raw fruit/vegetables and intestinal parasite

**Fig. 8:**
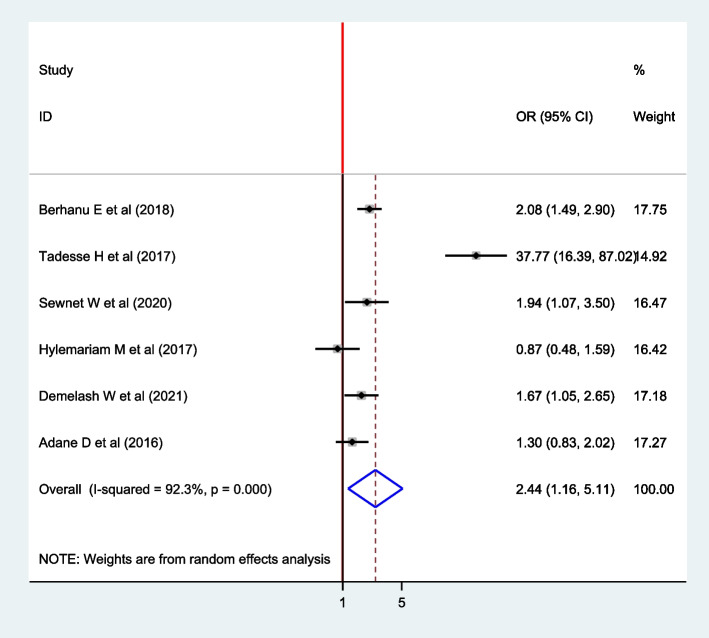
The Forest plot for the pooled association between habit of eating raw fruits and intestinal parasite infection among pregnant women in East Africa, 2021

Six articles were included in the analysis to estimate the pooled effect of a habit of eating raw fruit/vegetables on the intestinal parasite among pregnant women [9, 11, 15–17, 19]. The overall pooled estimate of a habit of eating raw fruit/vegetables revealed that there is an association between eating raw fruit/ vegetables and intestinal parasites (OR: 2.44; 95% CI: 1.16, 5.11). The pooled result revealed that intestinal parasite infection was 2.44 times higher among women who have a habit of eating raw fruits/vegetables compared to their counterparts. The random-effect model was used since heterogeneity was exhibited (I^2^ = 92.3%, *P* =  < 0.001) (Fig. 8).

### Association between educational status and intestinal parasite among pregnant women

**Fig. 9:**
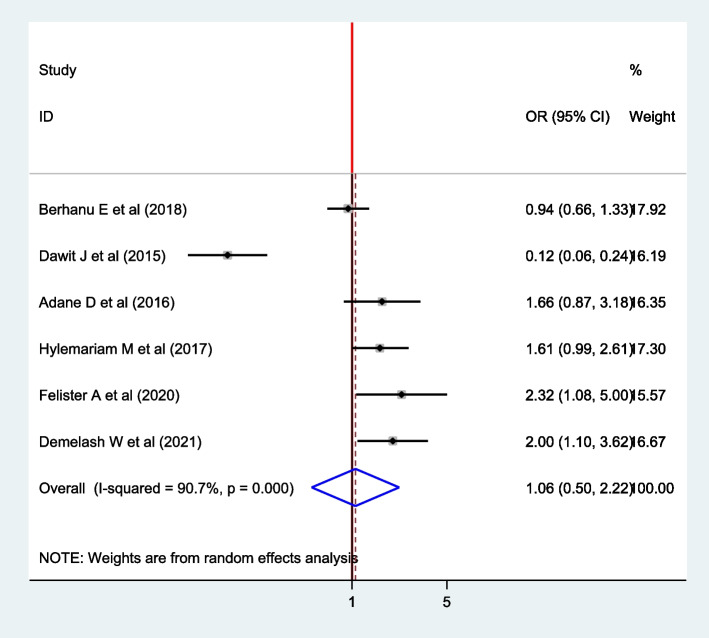
The forest plot for the pooled association between educational status and intestinal parasite infection among pregnant women in East Africa, 2021

Six articles were involved in the analysis to estimate the pooled effect of educational status and intestinal parasites among pregnant women [4, 9, 11, 16, 17, 24]. The overall pooled estimate of educational status revealed that there is no association between educational status and intestinal parasite (OR: 1.06; 95% CI: 0.50, 2.22). The random-effect model was used since heterogeneity was exhibited (I^2^ = 90.7%, *P* =  < 0.001) (Fig. 9).

### Association between barefooted and intestinal parasite among pregnant women

**Fig. 10:**
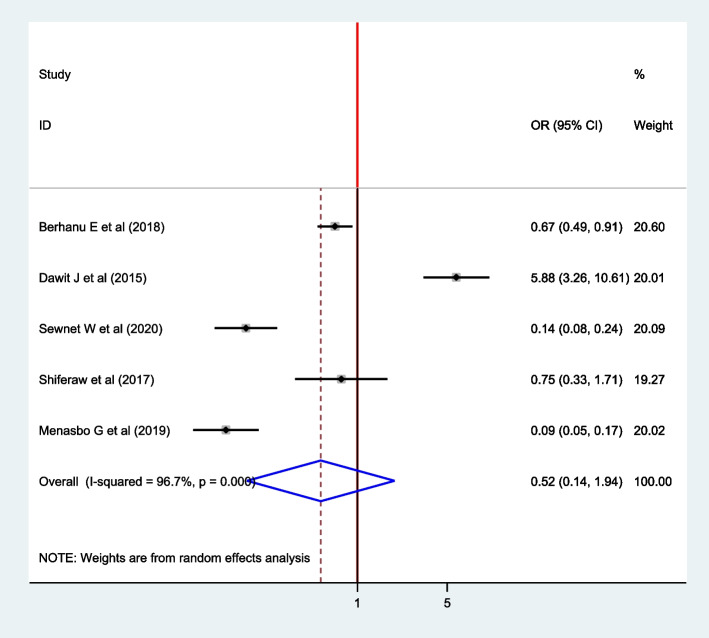
The Forest plot for pooled association between barefooted and Intestinal parasite infection among pregnant women in East Africa, 2021

Five articles were included to assess the pooled effect of barefooted and intestinal parasites among pregnant women [4, 9, 15, 20, 22]. From these, only one article showed a positive association between barefooted and intestinal parasite infection among pregnant women [4]. The pooled estimate effect revealed that there is no association between barefooted and intestinal parasites among pregnant women (OR: 0.52; 95% CI: 0.14, 1.94). The random-effect model was used since heterogeneity was exhibited (I^2^ = 96.7%, *P* =  < 0.001) (Fig. 10).

### Association between a habit of eating soil and intestinal parasite among pregnant women

**Fig. 11:**
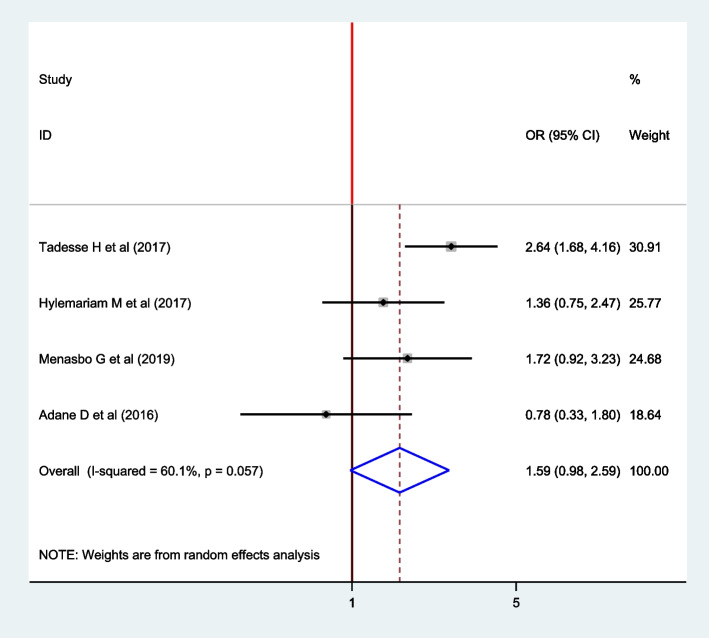
The Forest plot for the pooled association between eating soil and intestinal parasite infection among pregnant women in East Africa, 2021

Four articles were included to assess the association between the habit of eating soil and intestinal parasite among pregnant women [16, 17, 19, 20] in which only one article showed a significant association between the habit of eating soil and intestinal parasite [19]. However, the pooled estimate effect revealed that there is no association between eating soil and intestinal parasite among pregnant women (OR: 1.59; 95% CI: 0.98, 2.59). The random-effect model was used for this finding (I^2^ = 60.1%, *P* =  < 0.057) (Fig. 11).

### Association between the source of water and intestinal parasite among pregnant women

**Fig. 12:**
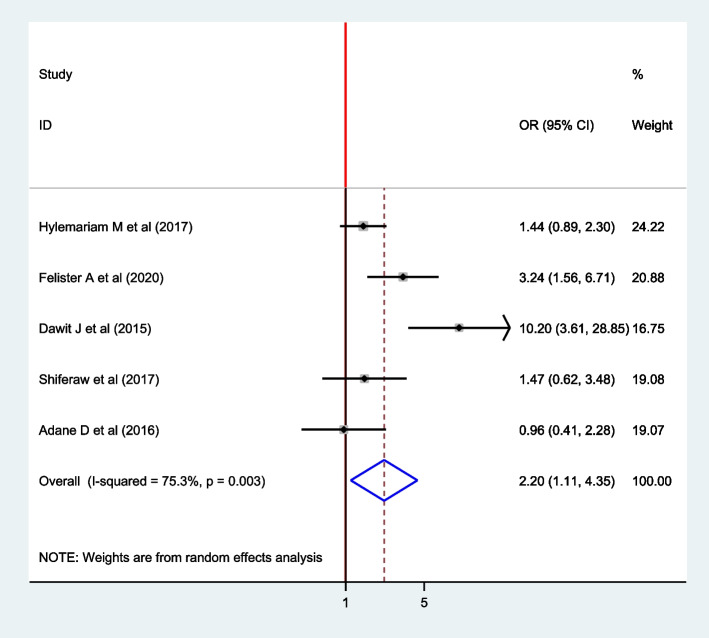
Forest plot for the pooled association between source of water and intestinal parasite infection among pregnant women in East Africa, 2021

Regarding the source of water, five articles, four from Ethiopia [4, 16, 17, 22] and one from Uganda [24] were included in the analysis. From these, two articles showed a significant association between the source of drinking water and intestinal parasite infection [4, 24] The pooled effect model showed a significant association between the source of water and intestinal parasite among pregnant women (OR: 2.20;95% CI: 1.11,4.35). The pooled result indicated that intestinal parasite infection was 2.20 times higher among women who drink unprotected water compared to their counterparts (Fig. 12). In this finding, random-effect model was used (I^2^ = 75.3%, *P*-value = 0.003).

### Association between Hand washing after toilet and intestinal parasite among pregnant women

**Fig. 13:**
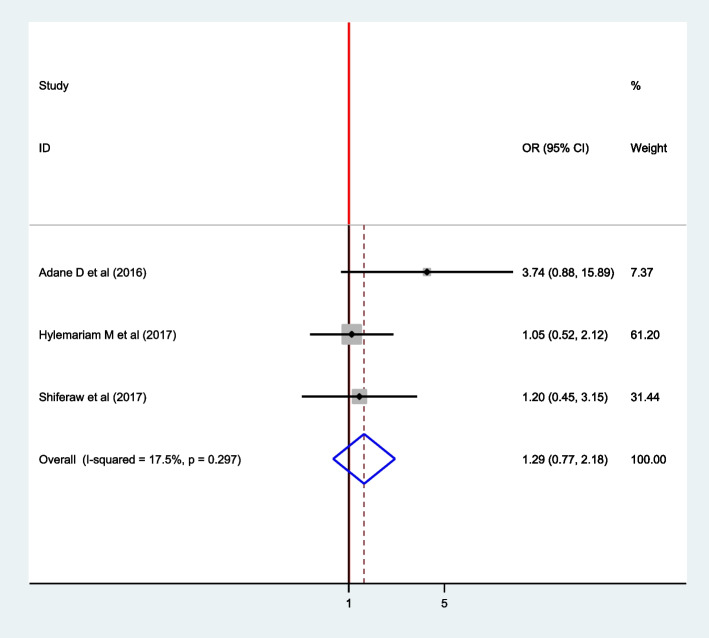
Forest plot pooled association between hand washing after toilet and intestinal parasite infection among pregnant women, 2021

To assess the association between handwashing after toilet and intestinal parasite infection, three articles were included [16, 17, 22] in which all of them didn’t show the association between handwashing after facility and intestinal parasite infection among pregnant women. The pooled effect model showed no significant association between handwashing after toilet and intestinal parasite infection among pregnant women OR: 1.29(95% CI: 0.77, 2.18) (Fig. 13). The fixed-effect model was used since no heterogeneity was exhibited (I^2^ = 17.5, *P* = 0.297).

## Discussion

The ultimate goal of preventing intestinal parasites among pregnant women is to reduce maternal and newborn morbidity and mortality. Consequently, effective preventive intervention and implementation approaches can be implemented if the burden and determinant factors are well identified. Thus, this systematic review and meta-analysis was conducted to estimate the pooled prevalence and determinant factors of intestinal parasites among pregnant women in East Africa.

The pooled prevalence of intestinal parasites among pregnant women in this systematic review and meta-analysis was 38.54 (28.77, 48.32). This is similar to findings of the studies conducted in India (42.67%) [25], Ghana (30.5%) [26], Ibadan Nigeria (43.4%) [27], Colombia (41%) [28], Southeast Nigeria (32.4%) [29], Tribhuvan University teaching hospital, Nepal (35%) [8], Ram Janaki Hospital, Janakpurdham, Nepal (42%) [30], and Cameroon (47.1%) [31]. The pooled prevalence in this study is lower than studies conducted in the Caribbean (66.2%) [32], Venezuela (73.9%) [33], Thai Burmese border, Southeast Asia (70%) [34], Guinea (81%) [35], Sub-Saharan Africa (66%) [36] and Indonesia (67.7%) [37]. The low prevalence in this review might be due to improvement in awareness creation for the community by health extension workers (in the case of East Africa) and working closely with the community on the risk of intestinal parasites and its prevention methods.

However, the pooled prevalence in this study is higher than studies conducted in Kasoa polyclinic, Ghana (14.3%) [38], Abeokuta, Nigeria (21.8%) [39], Benin (2.2%) [40], Enugu State, Nigeria (16.3%) [41], Osun State, Nigeria (12%) [42], and Edo State, Nigeria (18.2%) [43]. The discrepancy might be a result of the difference in access to health facilities and the socio-demographic characteristics of the study populations.

In this study, variables like residing in rural areas, availability of latrine, eating raw fruits/vegetables, and sources of water as unprotected sources show statistically significant associations with the burden of intestinal parasites among pregnant women. However, having the habit of eating soil, educational status, hand washing after toilet, and being barefooted had no statistical association with the burden of intestinal parasites among pregnant women.

The pooled effect of this systematic review and meta-analysis showed a positive association between rural residence and intestinal parasites among pregnant women. Pregnant women who come from rural areas were 3.75 times more likely to have intestinal parasites compared to women from Urban. This is consistent with studies conducted in a tertiary multispecialty teaching hospital in Mumbai, India [25] and a systematic review and meta-analysis conducted on the global prevalence of intestinal parasitic infections and associated risk factors in pregnant women [7]. This may be because, pregnant women who reside in rural areas are uneducated, less knowledgeable about the risk and preventive mechanisms of intestinal parasites, practice poor personal hygiene, use contaminated water, and mostly walk barefoot. All this can predispose them to intestinal parasites.

The pooled effect of the meta-analysis showed that the absence of latrine is positively associated with intestinal parasites. Pregnant women who have no latrine were 2.94 times more likely to have intestinal parasites compared to women who have a latrine. This is in line with studies conducted in a tertiary multispecialty teaching hospital in Mumbai, India [25], Kasoa Polyclinic, Ghana, in which pregnant women who shared a toilet facility had 2.78-fold greater odds of intestinal parasite infections compared to those who owned a toilet facility [38], and Abeokuta, Nigeria [39]. This may be because, in the absence of latrine, there is open defecation, which can contaminate food and water that pregnant women consume, and finally, predispose them to intestinal parasites.

In this review, the pooled estimate effect revealed that there is an association between eating raw fruits/vegetables and intestinal parasite infection among pregnant women. This finding is supported by a study conducted in India in which eating raw fruits/vegetables is a risk factor for intestinal parasites during pregnancy [25]. This is because eating raw fruits and vegetables is closely associated with helminthic diseases due to the high probability of the presence of infective parasitic ova in fruits/vegetables, which can predispose pregnant women to infection with intestinal parasites.

The pooled effect model of this systemic review and meta-analysis showed a significant association between the source of water and intestinal parasites among pregnant women. Pregnant women who use unprotected water sources were 2.2 times more likely to have intestinal parasites compared to women who use protected water sources. This is in line with the studies conducted in India [25], Ghana [26], and Southeast Nigeria [29]. This can be justified that unprotected water sources are prone to contamination with different wastes and pathogenic organisms like cysts of protozoon species and eggs of worms which are commonly transmitted to pregnant women by drinking contaminated water. However, in this review, the pooled estimate effect indicated that there is no statistical association between eating soil, educational status, being barefooted, and hand washing after the toilet.

### Strength and limitation of the study

Various databases and digital libraries were extensively searched for both published and unpublished articles. However, this review is not without limitations; as primary studies included in this review were cross-sectional in design; it is difficult to conclude the temporal relationship between the intestinal parasite infection and its determinants. Besides, this review included only articles published in the English language; articles published in another language might be excluded.

## Conclusion

The current systematic review and meta-analysis findings indicated that more than one-third of pregnant women in East Africa had intestinal parasite infection. This indicates that the burden of intestinal parasite infection among this group was high. Residing in rural, the absence of latrine, having the habit of eating raw fruits/vegetables, and using unprotected sources of drink water were the identified factors associated with Intestinal parasite infection among pregnant women. Therefore, efforts should be made in deworming pregnant women at the community and institutional level by stakeholders to reduce the burden of intestinal parasite infections and related complications.

## Data Availability

The data used for this study are presented within the manuscript.

## Abbreviations

IPI: Intestinal Parasite Infection
AOR: Adjusted Odds ratio
CI: Confidence Interval
JBI: Joanna Briggs Institute
WHO: World Health Organization
